# Construction of a genetic map for *Theileria parva*: Identification of hotspots of recombination

**DOI:** 10.1016/j.ijpara.2011.01.001

**Published:** 2011-05

**Authors:** Frank Katzer, Regina Lizundia, Daniel Ngugi, Damer Blake, Declan McKeever

**Affiliations:** a Royal Veterinary College, University of London, Hawkshead Lane, North Mymms, Hatfield, Hertfordshire AL9 7TA, UK; b Moredun Research Institute, Pentlands Science Park, Penicuik, Midlothian EH26 0PZ, UK

**Keywords:** *Theileria parva*, Recombination, Marikebuni, Muguga, Hotspot, Genetic map

## Abstract

The tick-borne protozoan parasite *Theileria parva* is the causal agent of East Coast Fever (ECF), a severe lymphoproliferative disease of cattle in eastern, central and southern Africa. The life cycle of *T. parva* is predominantly haploid, with a brief diploid stage occurring in the tick vector that involves meiotic recombination. Resolved genetic studies of *T. parva* are currently constrained by the lack of a genome-wide high-definition genetic map of the parasite. We undertook a genetic cross of two cloned isolates of *T. parva* to construct such a map from 35 recombinant progeny, using a genome-wide panel of 79 variable number of tandem repeat markers. Progeny were established by in vitro cloning of cattle lymphocytes after infection with sporozoites prepared from *Rhipicephalus appendiculatus* ticks fed on a calf undergoing a dual infection with the two clonal parental stocks. The genetic map was determined by assigning individual markers to the four chromosome genome, whose physical length is approximately 8309 kilobasepairs (Kb). Segregation analysis of the markers among the progeny revealed a total genetic size of 1683.8 centiMorgans (cM), covering a physical distance of 7737.62 Kb (∼93% of the genome). The average genome-wide recombination rate observed for *T. parva* was relatively high, at 0.22 cM Kb^−1^ per meiotic generation. Recombination hot-spots and cold-spots were identified for each of the chromosomes. A panel of 27 loci encoding determinants previously identified as immunorelevant or likely to be under selection were positioned on the linkage map. We believe this to be the first genetic linkage map for *T. parva.* This resource, with the availability of the genome sequence of *T. parva*, will promote improved understanding of the pathogen by facilitating the use of genetic analysis for identification of loci responsible for variable phenotypic traits exhibited by individual parasite stocks.

## Graphical abstract

**Graphical abstract:**

**Figure:**
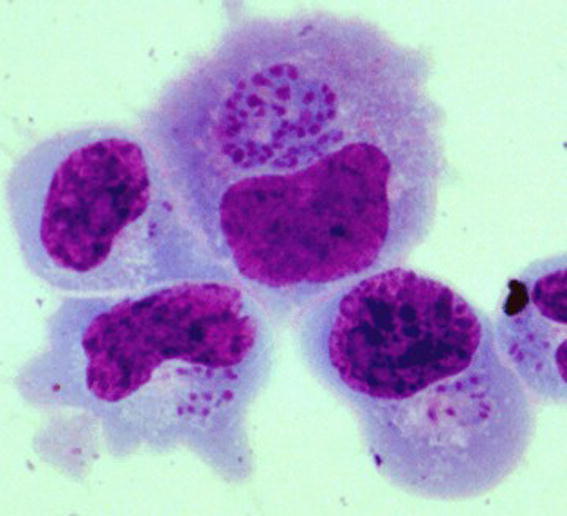

**Research highlights:**

► This is the first genetic map for *Theileria parva*. ► Thirty five independent recombinant progeny clones were genotyped using 79 VNTR markers. ► A total of 10 hot spots and 13 cold spots of recombination were identified. ► The average genome-wide recombination rate was relatively high (0.22 cM Kb^−1^). ► Relatively large genetic size (1683.8 cM) compared with other apicomplexa.

## 1 Introduction

*Theileria parva* is a tick-borne parasite of cattle that infects and transforms bovine lymphocytes, causing a severe lymphoproliferative disease known as East Coast Fever (ECF). Transmitted by the brown ear tick *Rhipicephalus appendiculatus*, the parasite is prevalent in eastern, central and southern Africa. The pathogenesis of ECF arises largely from the invasion of lymphoid and non-lymphoid tissues with proliferating infected lymphoblasts, and susceptible animals normally die within 3 weeks of infection. With an estimated 24 million cattle at risk of infection (Mukhebi et al., 1992) the disease is a major constraint to livestock productivity in the region. The life cycle of *T. parva* is similar to that of other apicomplexa and involves obligate developmental stages in both mammalian and vector hosts (Mehlhorn and Shein, 1984). It is predominantly haploid, with only a brief diploid phase in the tick. Cattle become infected by inoculation of sporozoite forms in tick saliva. Sporozoites invade host lymphocytes and differentiate to multinucleate schizont forms, which transform the infected cell to a state of uncontrolled proliferation. The parasite replicates in synchrony with the dividing lymphocyte and, by associating with the mitotic spindle (Hulliger et al., 1964), ensures transmission of infection to each daughter cell. With the progress of infection, the parasite ceases to divide in a proportion of infected cells and differentiates to uninucleate merozoites. These are released from the dying cell and invade erythrocytes, where they develop into tick-infective piroplasms. Cattle that recover from infection are almost invariably long-term carriers of these forms. Upon ingestion by a subsequent feeding tick, piroplams give rise to macro- and micro-gametes, which undergo syngamy in the gut lumen to yield diploid zygotes. These invade gut epithelial cells and undergo further differentiation, including meiotic division, to form motile kinetes, which migrate to the tick salivary gland. There they undergo further nuclear division, ultimately giving rise to cattle-infective uninucleate sporozoites. We have recently provided evidence for substantial genetic recombination between *T. parva* strains during passage through *R. appendiculatus* ticks (Katzer et al., 2006). Cattle can be immunised against *T. parva* by simultaneous inoculation of live sporozoites and long-acting formulations of oxytetracycline, a process known as ‘infection and treatment’ (Radley et al., 1975). There is strong evidence that the resulting protection is mediated by parasite-specific major histocompatibility complex (MHC) class I-restricted cytotoxic T lymphocytes (CTLs), which eliminate schizont-infected lymphoblasts (McKeever et al., 1994). The response is highly specific and therefore vulnerable to breakthrough by heterologous strains. Importantly, because it does not engage until the schizont parasitosis is established, the CTL response does not prevent infection, even after challenge with the immunising strain (Morrison et al., 1987). We have recently observed that, although CTLs impose considerable numerical attrition on targeted parasitised lymphoblasts in vivo, a proportion of parasites nonetheless emerge as piroplasms in the erythrocyte compartment. Responses are almost invariably restricted by one or other of the parental MHC haplotypes and generally focus almost entirely on a single antigenic determinant (MacHugh et al., 2009). In addition, the specificity of the response varies with MHC haplotype, suggesting that individual cattle in an outbred herd are likely to target distinct parasite components. We have shown that *T. parva* CTL antigens reassort during recombination in the tick (Katzer et al., 2006) and raised the possibility that this could result in evasion of such a tightly focussed effector response. In the face of immune selection, such an eventuality might be anticipated to result in augmented recombination of CTL antigenic loci over evolutionary time. In both yeast and human genomes, recombination occurs predominantly at specific regions known as hot spots (Petes, 2001; McVean et al., 2004), which can vary considerably in size (from hundreds of bp to tens of megabases (Mb)). A segment of DNA that undergoes recombination at the genome average rate can also be considered a hot spot if it is embedded in a very “cold” region of the genome. Although evidence for genetic crossover of *T. parva* in the tick has been obtained by satellite marker analysis (Katzer et al., 2006) and by tracking the segregation of several polymorphic sequences (Morzaria and Young, 1993), the extent of recombination and the location of hotspots across the genome remain to be determined. The recent availability of the *T. parva* genome sequence (Gardner et al., 2005) facilitated the establishment of a genome-wide panel of satellite markers for high-resolution genotyping of parasite populations. We have exploited these developments to undertake a broad genotypic analysis of a set of recombinant *T. parva* clones and their parents, with the aim of constructing, for the first time, a genetic map of the parasite. We draw on the information provided to evaluate the evidence for selection arising from bovine immunity and from other sources.

## 2 Materials and methods

### 2.1 Parasite populations

The study focused on two cloned stocks of *T. parva*, both of which were generated as described by Morzaria et al. (1995). Clone 3308 was derived from the Muguga (3087) stock of *T. parva*, while clone 4210 originated from the Marikebuni (3014) stock. The origins of these stocks have been described previously (Morzaria et al., 1995).

### 2.2 Animal infections

A male Boran (*Bos indicus*) calf was infected by s.c. inoculation behind the left and right parotid lymph nodes with sub-lethal quantities of sporozoites of both 3308 and 4210 clones. Doses were calculated to provide similar piroplasm parasitaemias for each clone on the basis of previous observations in vivo*.* The progress of infection was monitored by daily evaluation of rectal temperature from day 5, and microscopic examination of needle aspirates of the draining lymph node for the presence of *T. parva* schizonts on alternate days after the first manifestation of fever. Levels of parasitaemia for each clone were monitored during infection by PCR amplification of DNA extracted from whole blood, using discriminatory satellite markers. Approximately 1000 *R. appendiculatus* nymphs enclosed in cloth bags were applied at regular intervals to each ear of the calf from day 9 p.i. to feed during the piroplasm parasitaemia. Upon detachment, engorged ticks were evaluated for relative levels of infection with each parasite clone by satellite marker analysis of a representative sample. Those that detached on day 21 p.i. produced the most equivalent ratio for the two genotypes, and were chosen for stabilate production. Nymphs were removed after engorgement and incubated for 4 weeks at 28 °C and 85% relative humidity to promote moulting. Moulted infected adult ticks were pre-fed on rabbits for 4 days to stimulate sporogony. They were then removed and surface-sterilized by sequential rinses in 5% chlorhexidine, 70% ethanol and antibiotics, and analysed for parasite infection. The stabilate (CTVM St102) was prepared by trituration of sterilized ticks essentially as described by Brown (1987). All animal experiments were conducted with the formal approval of the institutional animal ethics committees. Standards of care and maintenance for experimental animals were in accordance with government and institutional guidelines.

### 2.3 In vitro infection and cloning

Peripheral blood mononuclear cells were isolated from defibrinated jugular venous bovine blood by flotation on Ficoll-Paque as described previously (Goddeeris and Morrison, 1988). Cells (10^7^) were resuspended in 1 ml of RPMI 1640 medium containing 2 mM glutamine, 5 × 10^–5^ M 2-mercaptoethanol, 100 IU/ml penicillin, 100 μg/ml streptomycin and 10% FCS (culture medium) and mixed with an equal volume of CTVM St102 diluted in culture medium, to obtain a multiplicity of infection of one tick equivalent/ml. The suspension was incubated for 2 h at 37 °C with occasional agitation before the addition of 8 ml culture medium and centrifugation at 200*g* for 10 min. Infected cells were then resuspended in culture medium at a density of 2.5 × 10^6^ cells/ml, dispensed into a 24-well plate in aliquots of 1 ml/well and incubated for 48 h at 37 °C in a humidified atmosphere of 5% CO_2_ and air. Cells were then harvested, assessed for viability by trypan blue exclusion and suspended in culture medium at a density of 10^5^ viable cells/ml. This suspension was used to seed 96-well plates in aliquots of 100 μl/well, with two plates each at 1 × 10^4^/well, 3 × 10^3^/well, 1 × 10^3^/well, 3 × 10^2^/well, and 1 × 10^2^/well. Each well then received 5 × 10^4^ irradiated (50 Gy) autologous peripheral blood mononuclear filler cells in 100 μl culture medium supplemented with 50% conditioned medium derived from established *T. parva*-infected lymphoblast cultures. Plates were incubated for 2–3 weeks at 37 °C in a humidified atmosphere of 5% CO_2_ in air and screened for the presence of single clones. One-third of each positive well was then harvested for the identification of hybrid clones using 24 polymorphic markers for preliminary screening by PCR analysis (Supplementary Table S1) and the residual culture was replenished with fresh medium. Unique clones were expanded for full genotyping as required and stored as live stabilates under liquid nitrogen.

### 2.4 Polymorphic markers and genotyping

#### 2.4.1 Satellite markers

The satellite marker panel described by Katzer et al. (2010) based on the original panel of Oura et al. (2003) was used, supplemented with an additional 13 polymorphic markers that were previously excluded due to cross-reaction with other *Theileria* spp. The additional markers were chosen on the basis that they discriminated between the parental clones and provided additional cover for the analysis due to their positions on the four chromosomes.

#### 2.4.2 Size polymorphisms

The satellite marker set was further enhanced by the inclusion of PCR primers that amplify polymorphic regions of four genes and/or open reading frames (ORFs) of *T. parva* and give rise to amplicon size polymorphisms that distinguish *T. parva* Muguga clone 3308 and *T. parva* Marikebuni clone 4210 (Katzer et al., 2006, 2007, 2010).

#### 2.4.3 PCR conditions and genotyping

The PCR conditions used in this study have been described previously (Katzer et al., 2006) and are essentially as outlined by Oura et al. (2003), except that the number of cycles was increased to 40, and Bioline (UK) *Taq* polymerase and a custom-made 10× PCR buffer (4.5 mM Tris–HCl (pH 8.8), 11 mM (NH4)_2_SO_4_, 4.5 mM MgCl_2_, 0.113 mg/ml BSA, 4.4 μM EDTA, 1.0 mM each of dATP, dCTP, dGTP, dTTP) purchased from ABgene (UK), were used. PCR products were separated on 2% Metasieve agarose (Flowgen, UK), visualised with ethidium bromide and photographed using a UV light box (BioRad, UK). The amplicon size at each marker locus was recorded and assigned a serial alphabetical code on the basis of the order in which it was first observed during the course of the analysis (Supplementary Fig. S1). Because the marker panel was first evaluated on a clone of the Muguga stock 273 of *T. parva*, alleles occurring at all loci in the Muguga population were designated A. Alleles occurring at loci in the Marikebuni population were designated B. The clone 407 for marker MS73 showed a novel amplicon product designated C, which may have arisen through replication slippage or possibly a recombination event.

### 2.5 Organisation of polymorphic markers into a genetic linkage map

The inheritance of 79 variable number of tandem repeat (VNTR) markers found to be polymorphic between the two parental *T. parva* cloned isolates was determined for all 35 independent recombinant progeny clones. These VNTR markers were organised into linkage groups and assigned to the four *T. parva* chromosomes using Map Manager QTX Software. For every marker, the parental alleles identified in each of the progeny clones were entered into an Excel spreadsheet. Data were then prepared for analysis with the Map Manager QTX software (version b.20) according to the instruction manual (Chmielewicz and Manly, 2002). Linkage groups were formed with a *P*-value of 0.001 using the “Make Linkage Groups” command in Map Manager with the Haldane mapping function. *P*-values in Map Manager indicate the probability of a Type 1 error; that is, the probability of a false positive linkage. The optimum marker order and genetic distance between markers in centiMorgans (cM) was calculated using the “Ripple” command. Physical map unit sizes were calculated by comparison of genomic marker location with the associated genetic distance (i.e. the physical distance corresponding to 1 cM). Haldane, Kosambi and Morgan map functions were tested on the data at the specified level of significance for comparative linkage criteria.

## 3 Results

### 3.1 Generation of a linkage map

**Fig. 1:**
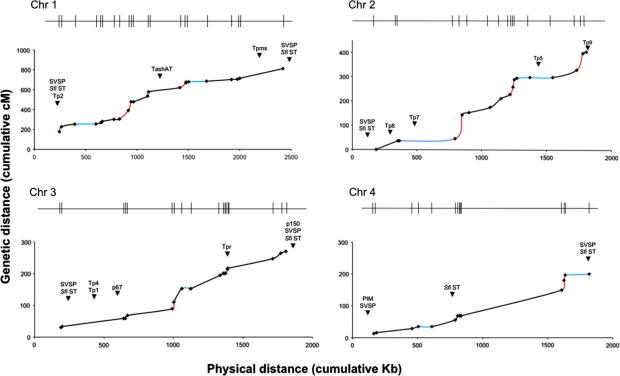
Plots indicating genetic distance as a function of physical distance for each chromosome of *Theileria parva*. *X*-axis, cumulative physical distance in kilobasepairs (Kb); *y*-axis, cumulative genetic distance in centimorgans (cM). Approximate positions of variable number of tandem repeat (VNTR) markers along the chromosomes are indicated on the bar above each graph. Intervals of high recombination rate (hot spots) are shown in red, while those of low recombination rate (cold spots) are shown in blue. Positions of loci encoding determinants previously identified as immunorelevant or likely to be under selection are indicated with black arrows. (For interpretation of the references to colour in this figure legend, the reader is referred to the web version of this article.)

A total of 35 recombinant *T. parva* clones were identified by screening 560 clonal cultures arising from the cross. These 35 unique clones were chosen from a preliminary screen using 24 polymorphic markers (six markers per chromosome) (Supplementary Table S1). All clones were genotyped using all 79 VNTR markers, providing inheritance patterns for each parent-specific marker (Supplementary Fig. S1). A genetic linkage map was constructed based on the segregation of each parent-specific marker within the recombinant progeny using the Map Manager QTX software. Haldane, Kosambi and Morgan map functions were tested on the data, but the linkage map described herein was informed by the Haldane function, which was in closest agreement with the consensus. Genetic distances, based on the number of recombination units between each marker, were expressed in cMs, which equate to a recombination frequency of 1%. The markers extended across the four chromosomes of the 8309 Kb *T. parva* genome at an average interval of 104 Kb (*σ* = 11.2) and therefore comprised an approximately uniform set. Linkage mapping generated four linkage groups and a total genetic size of 1683.8 cM, representing a physical distance of 7737.62 Kb (93% of the genome; Table 1; Fig. 1) covered by the marker set. Comparison of marker mapping among the recombinant progeny identified 434 crossover events, providing an average of 56 crossover events per Mb of mapped genome. Crossover frequency varied considerably between the chromosomes, ranging from 49 in chromosome 4 to 151 in chromosome 1. However, crossover frequency correlated with neither genetic nor physical mapped chromosome size.

### 3.2 Genome-wide recombination parameters

The physical rate of recombination was calculated by comparing the genetic and physical distance between each mapped marker (cM Kb^−1^). Accordingly, the average rate of recombination was approximately 0.22 cM Kb^−1^, although there was considerable variation within and between chromosomes (Table 1). The highest rate of recombination was observed for chromosome 1, at 4.61 cM Kb^−1^. The presence of hot spots and cold spots of recombination, defined here as at least five times higher or lower than the genome-wide average (i.e. ⩾1.05 and ⩽0.04 cM Kb^−1^) respectively, was evaluated across the four chromosomes. A total of 10 hot spots and 13 cold spots were identified (Table 1).

### 3.3 Candidate gene association with recombination rate

A panel of 27 genetic loci that encode proteins previously identified as immunorelevant or annotated as likely to be under selection were aligned with the linkage map (Fig. 1, Table 2). Allocation of the relevant rate of recombination to each locus position revealed two loci, comprising the *T. parva* repeat (Tpr) locus on chromosome 3 and the TashAT homologue gene cluster on chromosome 1, to be located within or between regions of high recombination activity (Table 2, Fig. 1). By contrast, the p67 locus and the loci encoding the CTL determinants Tp5 and Tp7 were found to be closely associated with regions of low recombination activity (cold spots) while the loci encoding Tp1, Tp2, Tp4, Tp8 and Tp9 were found in regions of average recombination rates. The SVSP loci 1–4, located in sub-telomeric regions of chromosomes 2, 1, 4 and 3, respectively, lay at the periphery of each respective linkage group, which limited our ability to assign recombination rates. The SVSP1 locus was found to be located close (a 166 bp interval) to a region of high recombination activity, but the remaining three SVSP loci were linked to regions with recombination parameters comparable to the genome average.

## 4 Discussion

Genetic maps have been constructed for a number of apicomplexan parasites, including *Eimeria tenella* (Shirley and Harvey, 2000), *Eimeria maxima* (Blake et al., 2011), *Plasmodium chabaudi chabaudi* (Martinelli et al., 2005), *Plasmodium falciparum* (Su et al., 1999) and *Toxoplasma gondii* (Sibley et al., 1992). We believe that the genetic map presented here is the first available for *T. parva*, representing the first non-*Plasmodium* map for parasites within the class Aconoidasida. Using a panel of 79 VNTR markers with known genomic locations, four linkage groups were constructed, consistent with the four chromosome *T. parva* genomic karyotype. At 1683.8 cM after a single meiotic opportunity, the genetic size of the *T. parva* genome is larger than has been described for any other apicomplexan parasite, despite its relatively small physical size of 8.3 Mb. For example, the estimated genetic size of the 23.3 Mb *P. falciparum* genome is 1556 cM, while *T. gondii*, at 63 Mb, has a predicted genetic size of only 592 cM. (current predicted genome sizes from EuPathDB, http://eupathdb.org/eupathdb/) (Su et al., 1999; Khan et al., 2005). The average genome-wide recombination rate for *T. parva* is therefore relatively high at 0.22 cM Kb^−1^ per meiotic generation (equivalent to an average map unit of 4.6 Kb cM^−1^), indicating that analysis of relatively few progeny can provide high mapping resolution. Pairwise comparison of the recombination rate between genetic markers revealed a high level of variation across the genome, both within and between chromosomes. Of the 10 recombination hot spots identified, only two were found to be closely associated with the end of a chromosome, although map coverage did not extend to the absolute end of any chromosome. Recent studies on the chromosome structure of *P. falciparum* have supported similar observations of variable recombination along a chromosome, but also reported elevated rates at chromosome ends, in contrast to our findings (Mu et al., 2005). Chromosome breakage occurs frequently in sub-telomeric genome regions and, in *P. falciparum,* genes encoding a number of immunodominant parasite proteins are located in these fragile chromosomal segments (Hernandez-Rivas et al., 1996). The sub-telomeric regions of *P. falciparum* contain arrays of repetitive sequences extending over 80–100 Kb (de Bruin et al., 1994). By contrast, sub-telomeric chromosome structure is much less complex in the *T. parva* genome. Indeed, many *T. parva* surface antigen-encoding loci are relatively conserved at the genus level and can be found distributed across the chromosomes, in contrast to the largely species-specific, sub-telomeric *Plasmodium* immunodominant antigens (Kuo and Kissinger, 2008). Genetic recombination can play a key role in the development of allelic diversity, through mechanisms such as gene duplication followed by rapid sequence divergence, and exon shuffling (Kuo and Kissinger, 2008). In addition, recombination can support rapid dissemination of an advantageous allele within a population under selection. For example, recombination could play a significant role in generating diversity at loci relevant to evasion of host immunity by *Theileria.* The identification of variable rates of recombination across the *T. parva* genome therefore prompted an assessment of the association between genes previously identified as immunorelevant and the local rate of recombination. Candidate antigens included Tp1, Tp2, Tp4, Tp5, Tp7, Tp8 and Tp9, previously shown to be targeted by the *T. parva*-specific CTL response (Graham et al., 2006; Katzer et al., 2006). Whilst polymorphism has been reported for several of the CTL-targeted antigens of *T. parva*, their coding sequences were all located within genomic regions characterised by low or neutral levels of recombination (Table 2). Other immunorelevant antigens include p67, a sporozoite surface antigen and vaccine candidate (Musoke et al., 1992; Nene et al., 1996). Despite recent reports that p67 in cattle-derived *T. parva* may be more polymorphic than previously thought (Sibeko et al., 2010), the p67 locus was found to be located in a region of relatively neutral recombination activity. *Theileria parva* antigens with high inter-specific non-synonymous/synonymous substitution (*d*_N_/*d*_S_) ratios, indicative of diversifying selection, include the polymorphic immunodominant molecule (PIM) and p150 (Toye et al., 1995; Skilton et al., 1998). Although the PIM and p150 loci occur in sub-telomeric regions of the *T. parva* genome, both also mapped to regions of relatively neutral recombination activity. Similarly, the *T. parva* merozoite surface antigen 1 (Tpms1), homologous to the highly polymorphic *Theileria annulata* merozoite surface antigen 1 (Tams1) (Katzer et al., 1998), is encoded sub-telomerically, in a region with a relative neutral recombination rate. Analysis of many Tams1 alleles from field isolates has provided evidence of high levels of intragenic recombination within this gene (Gubbels et al., 2000). The observation that Tpms1 locates to a region with a relatively neutral recombination frequency may reflect differences between these closely related parasites in recombination rates for this locus. However, this does not explain why other *T. parva* genes encoding proteins seen by the host immune response occur in regions with similarly low recombination frequencies. It is possible that these observations reflect a limited requirement for the parasite to generate diversity in these regions. Indeed, the extent to which the bovine immune response imposes selection on *T. parva* has been questioned previously, on the basis that protective cellular responses fail to prevent the emergence of tick-infective piroplasms (Katzer et al., 2007) and the observation that many of the identified antigenic targets of the response are conserved (McKeever, 2009). It has been argued that polymorphism observed in some immune determinants of the parasite arises from its evolution in the buffalo prior to its establishment in cattle (McKeever, 2009). Comparison of the genome sequences of *T. parva* and *T. annulata* has revealed strong conservation of synteny outside of sub-telomeric regions, with only a few inversions of small sequence blocks and no inter-chromosomal rearrangements (Pain et al., 2005). Exceptions corresponded to the insertion or deletion of genes and usually involved members of large gene families, most notably within the Tpr locus. The location of the Tpr locus within a recombination hot spot in these studies is consistent with these findings and the notion that the complex Tpr protein domain structure appears likely to support the rapid generation of genetic diversity (Gardner et al., 2005). Other *T. parva* gene families associated with diversifying selection based upon inter-species *d*_N_/*d*_S_ values include the TashAT/TashHN homologues, and the SVSP and *Sfi* sub-telomeric genes (Weir et al., 2010). Conservation of the *T. annulata* TashAT/TashHN gene family has recently been found at the intra-species level, despite the observation of elevated inter-species diversity (Weir et al., 2010). Interestingly, the *T. parva* TashAT/TashHN homologue locus mapped to the region containing the three hot spots with the highest observed recombination rates in this study. The *T. parva* SVSP and *Sfi* sub-telomeric-related genes are located, respectively, in eight and seven discrete sub-telomeric regions of all four chromosomes (Schmuckli-Maurer et al., 2009). The absence of genetic markers lying outside these gene arrays prevented determination of their associated recombination rates, although the neighbouring mapped regions exhibited relatively neutral or low levels of recombination. The SVSP gene family has recently been reported to present above average *d*_N_/*d*_S_ ratios but was found to be under neutral selection with the *T. annulata* genome (Weir et al., 2010), thus supporting the apparent absence of high recombination rates suggested by our study. Nonetheless, the resolution of this genetic map is limited by the density of markers and, to a lesser extent, the number of available clones. Some areas of chromosomes 2, 3 and 4 present a low density of markers (e.g. a region of ∼772 Kb between markers MS44 and MS45 in chromosome 4 is not covered by any marker). The resolution of a genetic map determines how precisely the position at which a recombination event takes place can be defined and it is ultimately limited by the density of polymorphisms. The position of an event cannot be resolved beyond the distance between the closest flanking markers. Increasing the number of independent clones may also improve map resolution, although the technically demanding nature of clone isolation imposed a practical limit in the present study. Considering the relatively low marker coverage for this study, it is possible that the frequency of hotspots of recombination has been underestimated in regions with low marker density, especially in situations where a hotspot might occur in close proximity to a cold spot.

Although our identification of hot and cold regions of recombination is based on a single genetic map, work with related apicomplexan parasites supports the reproducibility of genetic mapping and associated relative rates of recombination (e.g. Su et al., 1999; Khan et al., 2005). Further, the validity of the designations is supported by the statistical stringency applied during map construction. The availability of a genetic map for *T. parva* now provides a valuable resource for phenotype-driven quantitative trait loci (QTL) mapping and complements the existing genome sequence assembly. Development of additional markers targeting hot spots of genetic recombination will refine the map and support identification of genes with the greatest potential for diversification. Progress towards the annotation of putative and hypothetical coding sequences residing within each recombination hot spot will inform development of novel anti-*Theileria* control strategies, while identification of regions within the *T. parva* genome with the highest and lowest potential for genetic recombination will help to maintain the long-term efficacy of these strategies.

## Figures and Tables

**Table 1:** Summary statistics for the *Theileria parva* genetic map.

Chromosome	No. markers	Mapped size (Kb)	Total cM	Crossovers	cM/Kb	Hot spots	Cold spots
1	24	2418.67	811.9	151	0.34	4	3
2	18	1814.33	401.7	92	0.22	3	3
3	21	1771.65	270.5	142	0.15	2	4
4	16	1732.98	199.7	49	0.12	1	3
Total	79	7737.62	1683.8	434	–	10	13
Average	20	1934.41	421.0	109	0.22	3	3

Kb, kilobasepairs; cM, centiMorgans.

**Table 2:** Annotation of 27 genetic loci encoding *Theileriaparva* antigens previously identified as immuno-relevant (or as likely to be under selection) with the genetic map.

Chromosome	Locus	Chromosomal location (bp)	Context	Inter-species Polymorphism	Intra-species Polymorphism for *T. parva*	Reference	RR^a^
1	Sfi ST-c1a	3039–17,805	Sub-telomeric	High *d*_N_/*d*_S_	No data	Weir et al. (2010)	–b
1	SVSP-c1a	18,028–30,312	Sub-telomeric	High dN/dS	No data	Weir et al. (2010)	–b
1	Tp2	122,521–123,539	Internal	Highly polymorphic	Polymorphic between stocks	Graham et al. (2006)	–b
						MacHugh et al. (2009)	
1	TashAThomologues	1270,087–1274,623	Internal	High dN/dS	No data	Weir et al. (2010)	h
1	Tpms	2180,721–2181,765	Sub-telomeric	High dN/dS	Polymorphic between stocks	Weir et al. (2010)	–
1	Sfi ST-c1b	2514,060–2529,823	Sub-telomeric	High dN/dS	No data	Weir et al. (2010)	–b
1	SVSP-c1b	2531,301–2533,997	Sub-telomeric	High dN/dS	No data	Weir et al. (2010)	–b
2	Sfi ST-c2a	3020–7096	Sub-telomeric	High dN/dS	No data	Weir et al. (2010)	C^b^
2	SVSP-c2a	7952–21,726	Sub-telomeric	High dN/dS	No data	Weir et al. (2010)	C^b^
2	Tp8	285,551–287,131	Sub-telomeric	Highly conserved	No data	Graham et al. (2006)	–
2	Tp7	496,298–499,293	Internal	Conserved gene family	No data	Graham et al. (2006)	C
2	Tp5	1541,091–1541,855	Internal	Highly conserved	No data	Graham et al. (2006)	C
2	SVSP-c2b (Tp9)	1955,382–1969,335	Sub-telomeric	High dN/dS	No data	Weir et al. (2010)	–b
3	SVSP-c3a + Sfi ST-c3a	2862–617,912	Subtelomeric + Internal	High dN/dS	No data	Weir et al. (2010)	–b
3	Tp4	425,934–428,528	Internal	Highly conserved	No data	Graham et al. (2006)	–
3	Tp1	471,175–472,945	Internal	Highly divergent	Polymorphic between stocks	Graham et al. (2006)	
						MacHugh et al. (2009)	–
3	p67	588,213–590,371	Internal	Polymorphic	Polymorphic between stocks	Sibeko et al. (2010)	–b
3	Tpr (at least 28 genes)	∼1300,000–1400,000	Internal	High dN/dS	Highly polymorphic,	Weir et al. (2010)	H
						Gardner et al. (2005)	
3	p150	1808,704–1813,769	Sub-telomeric	Polymorphic	Polymorphic between stocks	Skilton et al. (1998)	–b
3	Sfi ST-c3b	1835,122–1836,330^c^	Sub-telomeric	High dN/dS	No data	Weir et al. (2010)	–b
3	SVSP-c3b	1836,591–1885,398^c^	Sub-telomeric	High dN/dS	No data	Weir et al. (2010)	–b
4	SVSP-c4a +	2938–174,574	Sub-telomeric	High dN/dS	No data	Weir et al. (2010)	–b
4	SfiST-c4a	796,343–797,246	Internal	High dN/dS	No data	Weir et al. (2010)	–b
4	PIM	85,086–86,644	Sub-telomeric	High dN/dS	Highly polymorphic	Toye et al. (1995)	–b
4	SVSP-c4b	1808,265–1821,765	Sub-telomeric	High dN/dS	No data	Weir et al. (2010)	c^b^
4	Sfi ST-c4b	1823,082–1832,984	Sub-telomeric	High dN/dS	No data	Weir et al. (2010)	c^b^

a Recombination rate annotation: H = in a hot spot, h = close to a hot spot, C = in a cold spot, c = close to a cold spot, – = no significant variation detected.

b Low density marker coverage may obscure selection.

c Based upon the sizes of the chromosome 3 contigs NW_876245 and NW_876244 plus the estimated missing sequence (Gardner et al., 2005).

## Appendix A Supplementary data

Supplementary data 1 Segregation of parental variable number of tandem repeat (VNTR) marker alleles among recombinant progeny of *Theileria parva.* VNTR marker positions on the four chromosomes are shown, together with the distribution of the parental alleles in each progeny clone. Muguga (Mu) clone 3308 alleles are depicted as A (yellow), while those of Marikebuni (Ma) clone 4210 are shown as B (orange). A novel amplicon product designated C (blue) was observed in clone 407 with marker MS73. This may have arisen through replication slippage or possibly a recombination event.
