# Supplementary material for: Construction of a genetic map for Theileria parva: Identification of hotspots of recombination
Source: Int J Parasitol. 2011 May;41(6-10):669–75. doi: 10.1016/j.ijpara.2011.01.001 (PMC3084458; doi:10.1016/j.ijpara.2011.01.001)
Supplement: Supplementary data 1 — Segregation of parental variable number of tandem repeat (VNTR) marker alleles among recombinant progeny of Theileria parva. VNTR marker positions on the four chromosomes are shown, together with the distribution of the parental alleles in each progeny clone. Muguga (Mu) clone 3308 alleles are depicted as A (yellow), while those of Marikebuni (Ma) clone 4210 are shown as B (orange). A novel amplicon product designated C (blue) was observed in clone 407 with marker MS73. This may have arisen through replication slippage or possibly a recombination event. [file mmc1.ppt]

## Slide 1
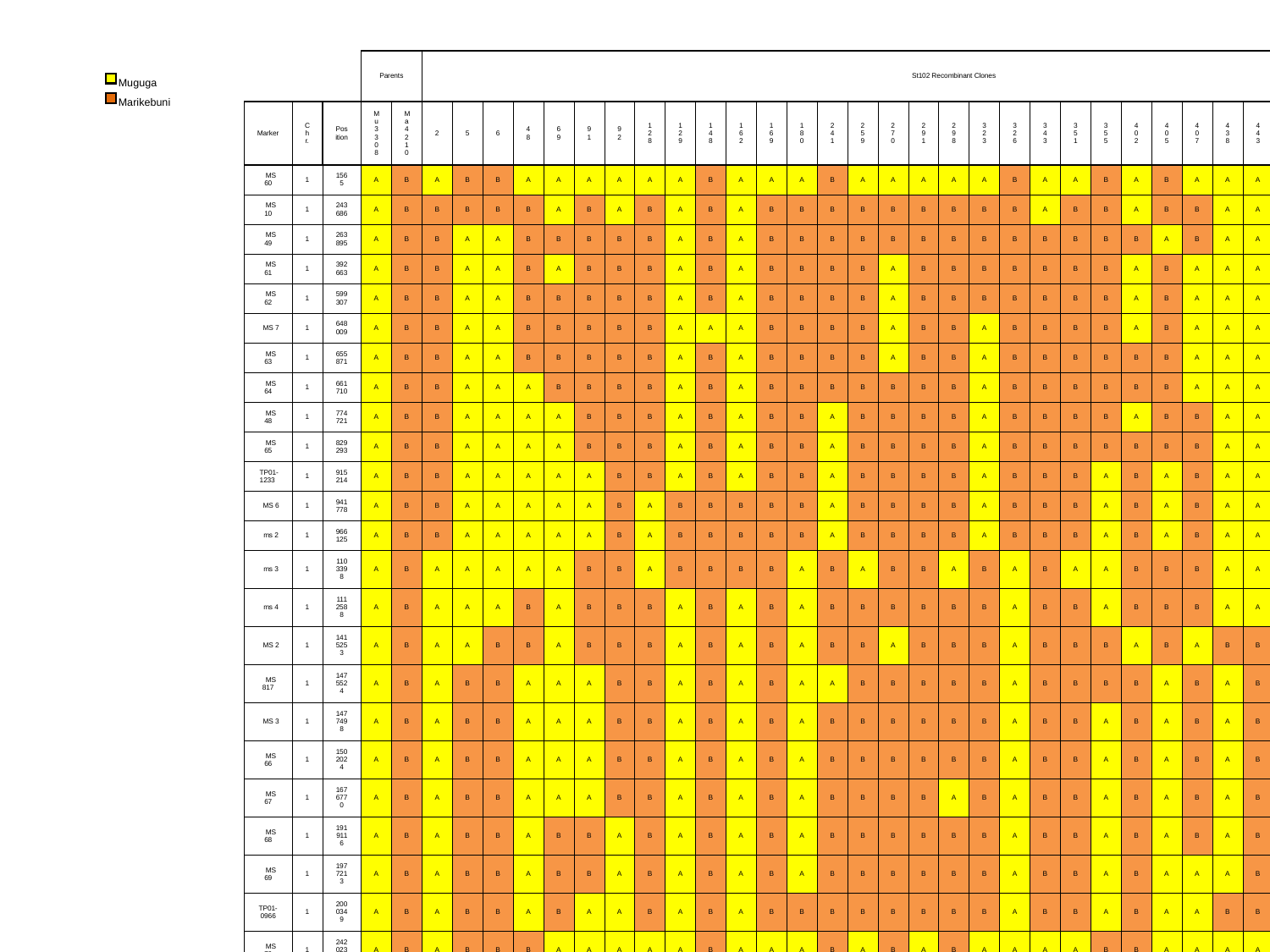

| | | | | | | | | | | | | | | | | | | | | | | | | | | | | | | | | | | | | | | | | | |
| --- | --- | --- | --- | --- | --- | --- | --- | --- | --- | --- | --- | --- | --- | --- | --- | --- | --- | --- | --- | --- | --- | --- | --- | --- | --- | --- | --- | --- | --- | --- | --- | --- | --- | --- | --- | --- | --- | --- | --- | --- | --- |
| | | | | Parents | | St102 Recombinant Clones | | | | | | | | | | | | | | | | | | | | | | | | | | | | | | | | | | | |
| | Marker | Chr. | Position | Mu 3308 | Ma 4210 | 2 | 5 | 6 | 48 | 69 | 91 | 92 | 128 | 129 | 148 | 162 | 169 | 180 | 241 | 259 | 270 | 291 | 298 | 323 | 326 | 343 | 351 | 355 | 402 | 405 | 407 | 438 | 443 | 451 | 456 | 471 | 479 | 522 | 529 | 550 | |
| | MS 60 | 1 | 1565 | A | B | A | B | B | A | A | A | A | A | A | B | A | A | A | B | A | A | A | A | A | B | A | A | B | A | B | A | A | A | A | A | B | A | A | B | A | |
| | MS 10 | 1 | 243686 | A | B | B | B | B | B | A | B | A | B | A | B | A | B | B | B | B | B | B | B | B | B | A | B | B | A | B | B | A | A | B | B | A | A | A | A | A | |
| | MS 49 | 1 | 263895 | A | B | B | A | A | B | B | B | B | B | A | B | A | B | B | B | B | B | B | B | B | B | B | B | B | B | A | B | A | A | A | B | B | B | A | B | A | |
| | MS 61 | 1 | 392663 | A | B | B | A | A | B | A | B | B | B | A | B | A | B | B | B | B | A | B | B | B | B | B | B | B | A | B | A | A | A | A | A | A | B | A | B | A | |
| | MS 62 | 1 | 599307 | A | B | B | A | A | B | B | B | B | B | A | B | A | B | B | B | B | A | B | B | B | B | B | B | B | A | B | A | A | A | A | A | A | B | A | B | A | |
| | MS 7 | 1 | 648009 | A | B | B | A | A | B | B | B | B | B | A | A | A | B | B | B | B | A | B | B | A | B | B | B | B | A | B | A | A | A | A | A | B | B | A | B | B | |
| | MS 63 | 1 | 655871 | A | B | B | A | A | B | B | B | B | B | A | B | A | B | B | B | B | A | B | B | A | B | B | B | B | B | B | A | A | A | A | A | B | B | A | B | B | |
| | MS 64 | 1 | 661710 | A | B | B | A | A | A | B | B | B | B | A | B | A | B | B | B | B | B | B | B | A | B | B | B | B | B | B | A | A | A | A | A | B | B | A | B | B | |
| | MS 48 | 1 | 774721 | A | B | B | A | A | A | A | B | B | B | A | B | A | B | B | A | B | B | B | B | A | B | B | B | B | A | B | B | A | A | B | A | B | B | B | B | B | |
| | MS 65 | 1 | 829293 | A | B | B | A | A | A | A | B | B | B | A | B | A | B | B | A | B | B | B | B | A | B | B | B | B | B | B | B | A | A | B | A | B | B | B | B | B | |
| | TP01-1233 | 1 | 915214 | A | B | B | A | A | A | A | A | B | B | A | B | A | B | B | A | B | B | B | B | A | B | B | B | A | B | A | B | A | A | B | A | B | B | B | B | B | |
| | MS 6 | 1 | 941778 | A | B | B | A | A | A | A | A | B | A | B | B | B | B | B | A | B | B | B | B | A | B | B | B | A | B | A | B | A | A | B | A | B | B | B | B | B | |
| | ms 2 | 1 | 966125 | A | B | B | A | A | A | A | A | B | A | B | B | B | B | B | A | B | B | B | B | A | B | B | B | A | B | A | B | A | A | B | A | B | B | B | B | B | |
| | ms 3 | 1 | 1103398 | A | B | A | A | A | A | A | B | B | A | B | B | B | B | A | B | A | B | B | A | B | A | B | A | A | B | B | B | A | A | A | A | B | B | B | B | A | |
| | ms 4 | 1 | 1112588 | A | B | A | A | A | B | A | B | B | B | A | B | A | B | A | B | B | B | B | B | B | A | B | B | A | B | B | B | A | A | A | A | A | B | B | A | B | |
| | MS 2 | 1 | 1415253 | A | B | A | A | B | B | A | B | B | B | A | B | A | B | A | B | B | A | B | B | B | A | B | B | B | A | B | A | B | B | A | A | B | B | A | A | A | |
| | MS 817 | 1 | 1475524 | A | B | A | B | B | A | A | A | B | B | A | B | A | B | A | A | B | B | B | B | B | A | B | B | B | B | A | B | A | B | A | B | B | B | B | A | A | |
| | MS 3 | 1 | 1477498 | A | B | A | B | B | A | A | A | B | B | A | B | A | B | A | B | B | B | B | B | B | A | B | B | A | B | A | B | A | B | A | B | B | B | B | A | A | |
| | MS 66 | 1 | 1502024 | A | B | A | B | B | A | A | A | B | B | A | B | A | B | A | B | B | B | B | B | B | A | B | B | A | B | A | B | A | B | A | A | B | B | B | A | A | |
| | MS 67 | 1 | 1676770 | A | B | A | B | B | A | A | A | B | B | A | B | A | B | A | B | B | B | B | A | B | A | B | B | A | B | A | B | A | B | A | A | B | A | B | A | A | |
| | MS 68 | 1 | 1919116 | A | B | A | B | B | A | B | B | A | B | A | B | A | B | A | B | B | B | B | B | B | A | B | B | A | B | A | B | A | B | A | A | B | A | B | B | A | |
| | MS 69 | 1 | 1977213 | A | B | A | B | B | A | B | B | A | B | A | B | A | B | A | B | B | B | B | B | B | A | B | B | A | B | A | A | A | B | A | A | B | A | B | B | A | |
| | TP01-0966 | 1 | 2000349 | A | B | A | B | B | A | B | A | A | B | A | B | A | B | B | B | B | B | B | B | B | A | B | B | A | B | A | A | B | B | A | A | B | A | B | B | A | |
| | MS 70 | 1 | 2420234 | A | B | A | B | B | B | A | A | A | A | A | B | A | A | A | B | A | B | A | B | A | A | A | A | B | B | A | A | A | A | A | A | B | B | B | A | A | |
| | MS 71 | 2 | 1623 | A | B | A | B | A | A | B | B | A | B | A | B | A | A | B | A | B | A | B | B | B | B | A | B | B | A | A | A | B | B | B | A | B | A | A | A | A | |
| | MS 72 | 2 | 184360 | A | B | A | B | A | A | B | B | A | B | A | B | A | A | B | A | B | A | B | B | B | B | A | B | B | A | A | A | B | B | B | A | B | A | A | A | A | |
| | MS 19 | 2 | 354329 | A | B | A | B | A | A | B | A | A | A | A | B | A | A | A | A | A | A | B | A | B | B | A | A | B | A | A | A | A | B | B | A | A | A | A | B | A | |
| | MS 18 | 2 | 366175 | A | B | A | B | A | A | B | A | A | A | A | B | A | A | A | A | A | A | B | A | B | B | A | A | B | A | A | A | A | B | B | A | A | A | A | B | A | |
| | MS 17 | 2 | 797000 | A | B | B | B | A | A | B | A | A | A | A | B | A | A | A | A | A | A | B | A | B | B | A | A | B | A | B | A | A | B | B | B | A | A | A | B | A | |
| | MS 12 | 2 | 852734 | A | B | A | B | B | A | A | A | B | A | A | B | A | B | A | A | A | A | A | A | A | A | B | A | B | A | B | B | A | B | A | B | B | B | A | A | B | |
| | MS 73 | 2 | 906540 | A | B | A | B | A | A | A | B | B | A | A | A | A | B | A | A | A | A | A | A | A | A | B | A | B | A | B | C | A | B | A | B | B | B | A | A | B | |
| | MS 74 | 2 | 1070539 | A | B | A | B | A | A | A | B | B | A | A | A | A | A | B | B | A | B | B | A | A | A | B | A | B | A | B | B | A | B | A | A | B | B | A | A | B | |
| | ms 7 | 2 | 1155482 | A | B | A | A | A | A | A | B | B | A | A | B | A | B | B | A | A | A | B | A | A | A | B | A | B | B | A | A | A | B | A | A | B | B | B | A | B | |
| | MS 11 | 2 | 1224954 | A | B | A | A | B | A | A | B | B | A | A | B | A | B | B | A | A | A | B | A | A | A | B | A | B | B | A | A | A | A | A | B | A | B | B | A | A | |
| | MS 14 | 2 | 1248020 | A | B | A | A | B | A | A | B | B | A | A | A | A | B | A | A | A | B | B | A | A | A | B | A | B | A | A | A | A | B | A | A | B | B | A | A | A | |
| | MS 16 | 2 | 1258364 | A | B | A | A | B | A | A | B | B | B | A | A | A | B | A | B | B | B | B | B | B | A | B | B | A | A | A | A | A | A | A | A | B | B | A | A | A | |
| | MS 75 | 2 | 1278718 | A | B | A | A | B | A | A | B | B | B | A | A | A | B | A | A | B | B | B | B | B | A | B | B | B | A | A | A | A | A | A | A | B | B | A | A | A | |
| | MS 76 | 2 | 1378981 | A | B | A | A | B | A | A | B | B | B | A | A | A | B | A | A | B | B | B | B | B | A | B | B | B | A | B | A | A | A | A | A | B | B | A | A | A | |
| | MS 77 | 2 | 1555168 | A | B | A | A | B | A | A | B | B | B | A | A | A | B | A | A | B | B | B | B | B | A | B | B | B | A | B | A | A | A | A | A | B | B | A | A | A | |
| | MS 78 | 2 | 1739970 | A | B | A | A | B | A | B | A | A | B | A | A | A | A | A | A | B | B | A | B | B | A | A | B | B | A | B | A | A | A | A | B | B | A | A | A | A | |
| | MS 15 | 2 | 1789213 | A | B | A | A | B | B | B | B | A | A | A | A | B | A | A | A | A | A | A | A | A | A | A | A | A | A | A | A | A | B | A | A | B | A | A | A | A | |
| | TP9 | 2 | 1815955 | A | B | A | A | A | B | B | B | A | A | B | A | B | A | A | A | A | A | A | A | A | A | A | A | A | A | A | A | A | B | A | A | B | A | A | A | A | |
| | MS 21 | 3 | 37058 | A | B | B | A | A | A | B | B | B | A | B | A | B | B | B | B | A | B | B | A | A | B | B | A | B | B | B | B | A | A | B | B | A | B | B | B | B | |
| | MS 22 | 3 | 189264 | A | B | B | B | A | B | A | B | A | A | B | A | B | B | B | B | A | B | B | A | A | B | B | A | A | B | A | B | B | A | B | A | A | B | B | B | B | |
| | ms 8 | 3 | 198664 | A | B | B | B | A | B | A | B | A | A | B | A | B | B | B | B | A | B | B | A | A | B | B | A | A | B | B | B | B | A | B | A | A | B | B | B | B | |
| | MS 23 | 3 | 640350 | A | B | B | B | A | A | A | B | A | A | B | B | B | B | B | B | A | B | B | A | A | B | B | B | A | A | A | B | A | A | B | B | A | B | B | B | B | |
| | MS 51 | 3 | 652255 | A | B | B | B | A | A | A | B | A | A | B | B | B | B | B | B | A | B | B | A | A | B | B | B | A | A | A | B | A | A | B | B | A | B | B | B | B | |
| | MS 24 | 3 | 668774 | A | B | B | B | A | A | A | B | A | A | B | B | B | B | B | B | A | B | B | A | A | B | B | B | A | B | A | A | B | A | B | B | A | B | B | B | B | |
| | MS 25 | 3 | 990577 | A | B | B | B | B | A | A | B | B | B | B | B | B | B | B | B | B | B | B | B | A | A | B | B | A | B | A | A | B | A | B | B | A | B | B | B | B | |
| | MS 52 | 3 | 1001983 | A | B | A | B | B | A | B | B | B | B | B | B | B | B | A | B | B | B | B | B | B | A | B | B | A | B | A | A | B | A | A | A | A | B | B | B | B | |
| | MS 53 | 3 | 1059716 | A | B | B | B | B | A | B | B | B | A | A | B | A | B | B | B | A | B | B | A | B | A | B | B | A | A | A | A | B | B | B | A | A | B | B | B | B | |
| | MS 54 | 3 | 1122077 | A | B | A | B | B | A | B | B | B | A | A | B | A | B | B | B | A | B | B | A | B | A | B | B | A | A | A | A | B | B | B | A | A | B | B | B | B | |
| | ms 9 | 3 | 1124644 | A | B | A | B | B | A | B | B | B | A | A | B | A | B | B | B | A | B | B | A | B | A | B | B | A | A | A | A | B | B | B | A | A | B | B | B | B | |
| | ms 10 | 3 | 1334942 | A | B | A | B | B | B | A | A | B | A | A | B | B | B | B | A | A | A | B | A | B | B | B | B | A | A | A | B | B | A | B | B | A | B | B | B | B | |
| | MS 56 | 3 | 1358970 | A | B | A | B | B | B | A | A | B | A | A | B | A | B | B | A | A | A | B | A | B | B | B | B | A | A | B | B | B | A | B | B | A | B | B | B | B | |
| | MS 31 | 3 | 1368422 | A | B | A | B | B | B | A | A | B | A | A | B | A | B | B | A | A | A | B | A | B | B | B | B | A | A | B | B | B | A | B | B | A | B | B | B | B | |
| | MS 30 | 3 | 1374488 | A | B | A | B | B | B | A | A | B | A | A | B | A | B | B | A | A | A | B | A | B | B | B | B | A | A | B | B | B | A | B | B | A | B | B | B | B | |
| | MS 29 | 3 | 1388664 | A | B | A | B | B | A | A | B | B | A | A | B | A | B | B | A | A | A | B | A | B | B | B | B | A | A | B | B | B | B | B | B | B | B | B | B | B | |
| | MS 28 | 3 | 1390242 | A | B | A | B | B | A | A | B | B | A | A | A | A | B | B | A | A | A | B | A | B | B | B | B | A | A | B | B | B | B | B | B | B | B | B | B | B | |
| | MS 312 | 3 | 1390246 | A | B | A | B | B | A | A | B | B | A | A | A | A | B | B | A | A | A | B | A | B | B | B | B | A | A | B | B | B | B | B | B | B | B | B | B | B | |
| | MS 55 | 3 | 1714018 | A | B | A | A | B | A | A | B | B | B | A | A | A | A | B | A | A | B | A | A | B | B | B | B | A | A | B | A | B | B | B | B | B | A | A | B | B | |
| | MS 27 | 3 | 1771747 | A | B | B | A | B | A | B | B | B | B | A | B | A | A | B | A | A | B | A | A | B | B | B | B | A | B | B | A | A | B | B | B | B | A | A | B | B | |
| | TP03-0861 | 3 | 1808704 | A | B | B | A | A | A | B | B | B | B | A | B | A | A | B | A | A | B | A | A | B | B | B | B | B | B | B | A | A | B | B | B | B | A | A | B | B | |
| | yPim F1 | 4 | 85625 | A | B | A | B | B | A | A | A | A | A | B | B | B | A | A | A | A | A | A | A | A | A | A | A | B | B | B | A | A | A | A | A | B | A | A | B | A | |
| | MS 33 | 4 | 163539 | A | B | A | B | B | A | A | A | A | A | B | B | B | A | A | B | A | A | A | A | A | A | A | A | B | B | B | B | A | B | A | A | B | A | B | B | A | |
| | MS 59 | 4 | 184070 | A | B | A | B | A | A | A | A | A | A | B | B | B | A | A | B | A | A | A | A | A | A | A | A | B | B | B | B | A | B | A | A | B | A | B | B | A | |
| | ms 11 | 4 | 457616 | A | B | A | B | A | A | B | B | A | A | B | B | A | A | A | B | A | A | A | A | A | B | A | A | B | B | B | B | A | B | A | A | B | A | B | B | A | |
| | MS 35 | 4 | 505891 | A | B | A | B | A | A | B | A | A | A | A | B | A | A | A | B | A | A | A | A | A | B | A | A | B | B | B | B | A | B | A | A | B | A | B | B | A | |
| | MS 38 | 4 | 609515 | A | B | A | B | A | A | B | A | A | A | A | B | A | A | A | B | A | A | A | A | A | B | A | A | B | B | B | B | A | B | A | A | B | A | B | B | A | |
| | MS 717 | 4 | 789129 | A | B | A | B | B | A | B | A | B | A | A | B | A | A | A | B | A | A | B | A | A | B | A | A | A | B | A | B | A | A | A | A | B | A | B | B | A | |
| | MS 39 | 4 | 807350 | A | B | A | B | B | A | B | A | B | A | A | B | A | A | A | B | A | B | B | A | A | B | A | A | B | B | B | B | A | B | A | A | B | A | B | B | A | |
| | MS 40 | 4 | 821953 | A | B | A | B | B | A | B | A | B | A | A | B | A | A | A | B | A | B | B | A | A | B | A | A | B | B | B | B | A | B | A | A | B | A | B | B | A | |
| | MS 42 | 4 | 825884 | A | B | A | B | B | A | B | A | B | A | A | B | A | A | A | B | A | B | B | A | A | B | A | A | B | B | B | B | A | B | A | A | B | A | B | B | A | |
| | MS 44 | 4 | 834099 | A | B | A | B | B | A | B | A | B | A | A | B | A | A | A | B | A | B | B | A | A | B | A | A | B | B | B | B | A | B | A | A | B | A | B | B | A | |
| | MS 45 | 4 | 1606841 | A | B | A | B | B | B | B | B | B | B | B | B | A | A | A | A | B | B | B | B | B | B | A | B | B | B | A | B | A | B | B | A | A | A | A | B | B | |
| | MS 58 | 4 | 1624149 | A | B | B | B | B | B | B | B | A | A | B | B | B | A | B | A | B | B | B | B | B | B | A | B | B | B | A | B | A | B | B | B | A | B | B | B | B | |
| | MS221B | 4 | 1633982 | A | B | B | B | B | B | B | B | A | A | B | B | A | A | B | A | B | B | B | A | A | B | A | A | B | B | A | B | A | A | B | B | A | B | B | B | B | |
| | MS221A | 4 | 1634557 | A | B | B | B | B | B | B | B | A | A | B | B | A | A | B | A | B | B | B | A | A | B | A | A | B | B | A | B | A | A | B | B | A | B | B | B | B | |
| | MS 46 | 4 | 1818602 | A | B | B | B | B | B | B | B | A | A | B | B | A | A | B | A | B | B | B | A | A | B | A | A | B | B | A | B | A | B | B | B | A | B | B | B | B | |
| | | | | | | | | | | | | | | | | | | | | | | | | | | | | | | | | | | | | | | | | | |
Muguga
Marikebuni
