# Supplementary material for: Construction of a genetic map for Theileria parva: Identification of hotspots of recombination
Source: Int J Parasitol. 2011 May;41(6-10):669–75. doi: 10.1016/j.ijpara.2011.01.001 (PMC3084458; doi:10.1016/j.ijpara.2011.01.001)
Supplement: Supplementary data 2 [file mmc2.doc]

**Supplementary Table S1.** Additional markers used for preliminary screening of recombinant clones.

| **Chromosome 1** | **Chromosome 2** | **Chromosome 3** | **Chromosome 4** |
| --- | --- | --- | --- |
| MS49 | MS18 | MS21 | yPimF1 |
| MS7 | MS17 | MS25 | MS33 |
| MS6 | ms7 | ms9 | ms11 |
| ms4 | MS14 | ms10 | MS39 |
| MS817 | MS16 | MS312 | MS45 |
| TP01-0966 | MS15 | MS27 | MS46 |
